# Cerebrospinal fluid MinION sequencing of 16S rRNA gene for rapid and accurate diagnosis of bacterial meningitis

**DOI:** 10.1016/j.jinf.2019.12.011

**Published:** 2020-04

**Authors:** Nguyen Thi Thu Hong, Ho Dang Trung Nghia, Tran Tan Thanh, Nguyen Phu Huong Lan, Nguyen Thi Han Ny, Nghiem My Ngoc, Vu Thi Ty Hang, Le Thi My Chau, Van Xuan Quynh, Le Thi Diem, Bui Thi Bich Hanh, Nguyen Ho Hong Hanh, Du Trong Duc, Dinh Nguyen Huy Man, James Campbell, Pham Kieu Nguyet Oanh, Jeremy Day, Nguyen Hoan Phu, Nguyen Van Vinh Chau, Guy Thwaites, Le Van Tan

**Affiliations:** a Oxford University Clinical Research Unit, Ho Chi Minh City, Vietnam; b Pham Ngoc Thach University of Medicine, Ho Chi Minh City, Vietnam; c Hospital for Tropical Diseases, Ho Chi Minh City, Vietnam; d Department of Medicine, Vietnam National University, Ho Chi Minh City, Vietnam; e Centre for Tropical Medicine and Global Health, Nuffield Department of Medicine, University of Oxford, Oxford, United Kingdom

**Keywords:** Streptococcus agalactiae, Meningitis, MinION, Nanopore, 16S rRNA

*Dear Editor*,

We read with interest recent articles in this journal regarding the utility of next-generation sequencing for the diagnosis bacterial meningitis.^1^^,^^2^ Bacterial meningitis causes substantial morbidity and mortality worldwide.^3^ Rapid identification of the microorganisms is essential for early initiation of appropriate antimicrobial therapy, thereby improving clinical outcome. Yet routine diagnostic methods fail to identify the bacteria in the majority of patients. Over the last decade, advanced sequencing technologies have greatly improved our capacity to detect the causative agents of infectious diseases in clinical samples.^4^^,^^5^ Of these, the single molecule real-time sequencing developed by Oxford Nanopore Technologies (ONT) is a promising tool for diagnostic setting because of its short turnaround time.

In late April 2019, a 59-year old seller of fish-noodles was referred to our hospital with a 1-day history of headache, fever and vomiting. He had a history of heavy alcohol use and hepatitis C infection, and had cirrhosis and diabetes mellitus. On admission, he was unconsciousness (a Glasgow Coma Scale of 8), with a body temperature of 40 °C, a blood pressure of 140/80 mmHg and neck stiffness. Initial Gram-stain and microscopy of CSF showed Gram-positive cocci, 8449 white cells/uL with 91% neutrophils, elevated protein and low glucose level, and high lactate concentration (Fig. 1A). Routine bacterial culture, plus *Streptococcus pneumoniae* and *S. suis* PCRs were all negative. He was diagnosed with bacterial meningitis, and given a combination of ceftriaxone (2 g/12 h) and dexamethasone (0.4 mg/kg/12 h). His clinical condition steadily improved. His second and third CSF samples became negative by Gram stain. The other CSF parameters also improved, except the glucose, which remained low (Fig. 1A). On day 20 of hospitalization, the patient suddenly became unconsciousness with fever. Brain magnetic resonance imaging showed bifrontal abscesses (Fig. 1B). After consulting a local neurosurgeon, aspiration of the brain abscesses was not advised and the patient was treated empirically with meropenem (2 g/8 h) and vancomycin (1 g/8 h). Due to continued diagnostic uncertainty, we performed 16S rRNA sequencing of the admission CSF, stored as part of an going clinical study (Supplementary Materials), using an established Sanger-sequencing based 16S rRNA method.^6^ Subsequently, analysis of the obtained sequences revealed evidence of *S. agalactiae* (Supplementary Figure 1). Given this new diagnostic result of the admission CSF and because the patient had recovered clinically, the patient was given 24 million units of penicillin G for every 4 h. After day 43 of hospitalization, all CSF parameters had normalised (Fig. 1A). Likewise, on CT scan the brain abscess was now significantly improved (Fig. 1C). The patient was discharged with full clinical recovery.

**Fig. 1:**
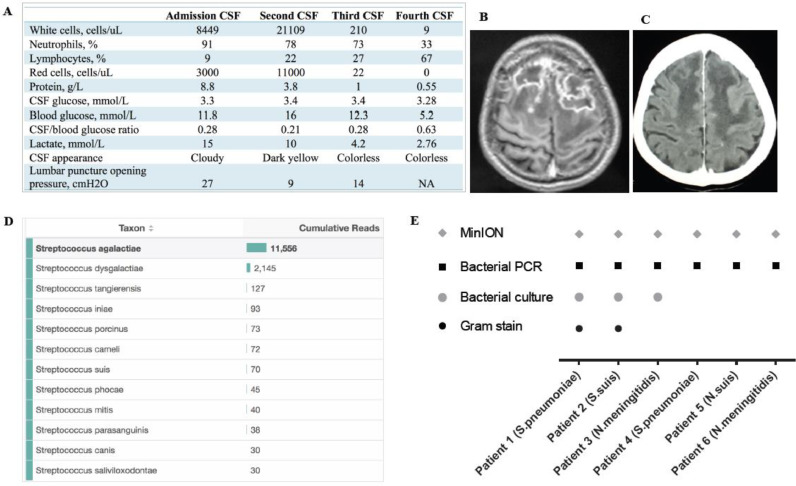
Clinical profile of the *S. agalactiae* patient, and result of CSF MinION sequencing of 16S rRNA gene. (A) results of CSF investigations over the course of illness; (B) MRI showing bifrontal brain abscesses; (C) follow-up CT scan performed on 5th June 2019 showing the improvement of the brain abscesses; (D) results of MinION sequencing of the admission CSF samples taken on 25th April 2019. A total of 14,848 reads were obtained after 100 min of the sequencing procedure, of which1,556 (78%) reads were successfully aligned *S. agalactiae*; (E) result of MinION sequencing of 16S rRNA gene analysis of the six additional CSF samples alongside routine diagnostic yields. The appearances of specific symbols indicate the success of the corresponding methods in detecting the pathogens in the tested samples. *Note to Figure 1:* The sampling dates of the second, third and fourth CSF samples were 27th April 2019, 17th May 2019, and 5th June 2019, respectively.

Additionally, MinION sequencing of complete 16S rRNA gene was retrospectively carried out on the extracted nucleic acid of the admission CSF yielded a total of 14,848 reads after 100 min of sequencing run. Of these, 11,556 reads (79%) were successfully aligned to *S. agalactiae* (Fig. 1D). The remaining reads were assigned to other Streptococcus species (mostly *S. dysgalacticiae* (*n* = 2.145, 14%)), likely attributed to a combination of the high level of sequence similarities of the 16S rRNA region between them and the sequencing errors introduced by the MinION systems. Analysis of sequencing data generated during the 25, 50 and 75 min of sequencing run time also yielded the same results (Supplementary Figure 2). Details about the MinION procedure are presented in Supplementary Materials.

To further assess of the utility of CSF MinION sequencing of 16S rRNA gene for the detection of bacterial meningitis pathogens, six CSF samples from patients with confirmed bacterial meningitis enrolled in the abovementioned clinical study were tested (Table 1). Analysis of the MinION reads obtained after two hours of the sequencing run showed that the majority of reads were correctly assigned to the corresponding bacterial species (*S. pneumoniae* and *S. suis)* or genus (*Neisseria*) found in the CSF samples by diagnostic work up of the clinical study (Fig. 1E and Table 1). Additional analysis of the obtained reads generated at two earlier time points (20 min and 60 min) of the sequencing run generated the same results (Table 1).

**Table 1:** Demographics and clinical outcome of the additional six patients included for MinION Nanopore sequencing analysis of 16S rRNA gene.

	Patient 1	Patient 2	Patient 3	Patient 4	Patient 5	Patient 6
**Demographics**						
Age (years)	33	65	23	29	53	41
Gender	Male	Male	Female	Male	Male	Female
Origin	BP	BT	BP	NT	BT	TN
**Illness day at enrollment (days)**	5	3	1	15	4	2
**Length of hospital stay (days)**	17	5	12	15^	13	15
**Clinical signs/symptoms**						
Body temperature ( °C)	37	38	38	37	37.2	37
Cranial nerve palsy	N	N	N	Y	N	N
Hemiplegia/paresis	N	N	N	N	N	N
Paraplegia/paresis	N	N	N	N	N	N
Tetraplegia/paresis	N	N	N	N	N	N
Generalized convulsions	N	N	N	N	N	N
Localized convulsions	N	N	N	N	N	N
Neck stiffness	Y	Y	Y	Y	Y	N
GCS at enrolment	14	14	13	13	9	12
**CSF examinations**						
CSF white cell counts	51,810	1609	3111	1126	16,744	4760
CSF neutrophils (%)	78	95	94	60	65	88
CSF lymphocytes (%)	22	5	6	40	35	12
CSF/blood glucose ratio	0.11	0.64	0.014	0.42	0.32	0.028
CSF lactate	11.4	9.21	12.45	5.82	13.94	15.62
Total protein	1.33	1.133	4.731	1.37	3.861	5.746
**Routine microbial investigations**						
ZN smear	ND	ND	Negative	Negative	ND	ND
India Ink stain	Negative	ND	Negative	Negative	ND	ND
Cryptococcal antigen test	ND	ND	ND	Negative	ND	ND
Gram stain	Gram-positive cocci	Gram-positive cocci	Negative	Negative	Negative	Negative
Bacterial culture	*S. pneumoniae*	*S. suis*	*N. meningitidis*	Negative	Negative	Negative
Bacterial PCR	*S. pneumoniae*	*S. suis*	*N. meningitidis*	*S. pneumoniae*	*S. suis*	*N. meningitidis*
**MinION 16S rRNA sequencing**						
20 min	*S. pneumoniae*	*S. suis*	*Neisseria*	*S. pneumoniae*	*S. suis*	*Neisseria*
1 h	*S. pneumoniae*	*S. suis*	*Neisseria*	*S. pneumoniae*	*S. suis*	*Neisseria*
2 h	*S. pneumoniae*	*S. suis*	*Neisseria*	*S. pneumoniae*	*S. suis*	*Neisseria*
**GCS at discharge**	15	14	15	14	14	15

**Notes to**Table 1: GCS: Glasgow Coma Score, BT: Ben Tre, BP: Binh Phuoc, TN: Tay Ninh, NT: Ninh Thuan, HCMC: Ho Chi Minh City, BM: bacterial meingitis, TBM: tuberculous meningitis; N: no, Y: yes; ND: not done.

Collectively, we report the first application of MinION sequencing of 16S rRNA gene to detect bacterial meningitis causing pathogens in CSF samples from a low and middle-income country. The assay was able to detect the bacterial causes in all of the seven tested CSF samples. Meanwhile, Gram stain and culture, the two most commonly used methods in clinical microbiology laboratories worldwide, were negative in 3/7 samples. (Fig. 1 and Table 1).

In addition to CSF samples described in the present study and a recent pilot study from Korea,^7^ successful detections of *Haemophilus influenzae* in sputum and *Campylobacter fetus* in culture materials by MinION sequencing of 16S rRNA have recently been reported.^8^ Together, the data suggest that MinION sequencing of 16S rRNA is a sensitive method for rapid and accurate detection of pan-bacterial pathogens, including unexpected microorganisms, in clinical samples. Additionally, the bacterial species information generated by the analysis of 16S rRNA sequences can be useful for disease surveillance and vaccine evaluation. Thus, the application of the method would be relevant for both patient management and epidemiological research. Indeed, to the best of our knowledge the present study represents the first report of *S. agalactiae* associated meningitis in Vietnam. Because the incidence of invasive diseases (including meningitis) caused by *S. agalactiae* has been reported with increased frequency in recent years,^9^ *S. agalactiae* should be considered as an important differential diagnosis for patients presenting with acute CNS infections in Vietnam.

Owing to the unavailability of the reagents at the time of patient admission, we were not able to perform real-time diagnosis using MinION sequencing on the collected CSF samples. However, same day diagnosis is theoretically achievable, because the current workflow takes 5 – 6 h to operate. Prospective study is urgently needed to assess its translational potential in the diagnosis of bacterial meningitis.

## The clinical study

Since September 2017, a prospective observational study aiming at exploring the utility potential of next-generation sequencing in patients presenting with central nervous system (CNS) infections has been conducted in the brain infection ward of the Hospital for Tropical Diseases (HTD) in Ho Chi Minh City, Vietnam. HTD is a tertiary referral hospital for patients with infectious diseases from southern provinces of Vietnam, serving a population of >40 million. Any patient (≥16 years) with an indication for lumbar puncture was eligible for enrolment. Patient was excluded if no written informed consent was obtained. As per the study protocol, CSF, plasma and urine samples were collected at presentation alongside demographic, meta-clinical data and results of routine diagnosis. After collection, all clinical specimens were stored at −80 °C until analysis.

The clinical study received approvals from the Institutional Review Board of the HTD and the Oxford Tropical Research Ethics Committee of the University of Oxford. Written informed consent was obtained from each study participant or relative (if the patient was unconsciousness).

## MinION sequencing of 16S rRNA

Sequencing of complete 16S rRNA gene was retrospectively performed using MinION Nanopore sequencer (ONT), following the manufacturer's instructions. In brief, amplification of the complete 16S rRNA gene and library preparation were carried out on extracted nucleic acid using 16S Barcoding Kit (SQK-RAB204, ONT) and primers (27F 5′-AGAGTTTGATCCTGGCTCAG-3′ and 1492R 5′-GGTTACCTTGTTACGACTT-3′), followed by the sequencing of the amplified product using R9.4 Flow cells (ONT). MinION reads were first basecalled using Albacore v2.1.7 (ONT), followed by demultiplexing using Porechop (https://github.com/rrwick/Porechop). Determination of bacterial genus/species composition in the obtained reads was then carried out using Epi2Me interface (Metrichor, Oxford, UK), a platform for cloud-based analysis of MinION data. Overall, the whole procedure of MinION sequencing of 16S rRNA gene takes 5–6 h to complete (Supplementary Figure 3).

## Declaration of Competing Interest

We, the author of the submitted manuscript declare that we do not have a commercial or other association that might pose a conflict of interest (e.g., pharmaceutical stock ownership, consultancy, advisory board membership, relevant patents, or research funding).

## Appendix Supplementary materials

Image, application 1

## References

[1] Guo L.Y., Li Y.J., Liu L.L., Wu H.L., Zhou J.L., Zhang Y. (2019). Detection of pediatric bacterial meningitis pathogens from cerebrospinal fluid by next-generation sequencing technology. J Infect.

[2] Zhang J.Z., Zheng P., Sun H.M., Dong J.J., Li S.L., Fan S.Y. (2019). Next-generation sequencing combined with routine methods to detect the pathogens of encephalitis/meningitis from a chinese tertiary pediatric neurology center. J Infect.

[3] Costerus J.M., Brouwer M.C., Bijlsma M.W., van de Beek D. (2017). Community-acquired bacterial meningitis. Curr Opin Infect Dis.

[4] Kai S., Matsuo Y., Nakagawa S., Kryukov K., Matsukawa S., Tanaka H. (2019). Rapid bacterial identification by direct pcr amplification of 16S rRNA genes using the MinION™ nanopore sequencer. FEBS Open Bio.

[5] Moon J., Kim N., Lee H.S., Shin H.R., Lee S.T., Jung K.H. (2017). Campylobacter fetus meningitis confirmed by a 16S rRNA gene analysis using the MinION nanopore sequencer, South Korea, 2016. Emerg Microbes Infect.

[6] Tytgat B., Verleyen E., Obbels D., Peeters K., De Wever A., D'Hondt S. (2014). Bacterial diversity assessment in antarctic terrestrial and aquatic microbial mats: a comparison between bidirectional Pyrosequencing and cultivation. PLoS One.

[7] Moon J., Kim N., Kim T.J., Jun J.S., Lee H.S., Shin H.R. (2019). Rapid diagnosis of bacterial meningitis by nanopore 16S amplicon sequencing: a pilot study. Int J Med Microbiol.

[8] Moon J., Jang Y., Kim N., Park W.B., Park K.I., Lee S.T. (2018). Diagnosis of haemophilus influenzae pneumonia by nanopore 16S amplicon sequencing of sputum. Emerg Infect Dis.

[9] Francois Watkins L.K., McGee L., Schrag S.J., Beall B., Jain J.H., Pondo T. (2019). Epidemiology of invasive group b streptococcal infections among nonpregnant adults in the united states, 2008-2016. JAMA Intern Med.

