# Supplementary material for: Cerebrospinal fluid MinION sequencing of 16S rRNA gene for rapid and accurate diagnosis of bacterial meningitis
Source: J Infect. 2020 Apr;80(4):469–96. doi: 10.1016/j.jinf.2019.12.011 (PMC7113840; doi:10.1016/j.jinf.2019.12.011)
Supplement: Supplementary file 1 [file mmc1.docx]

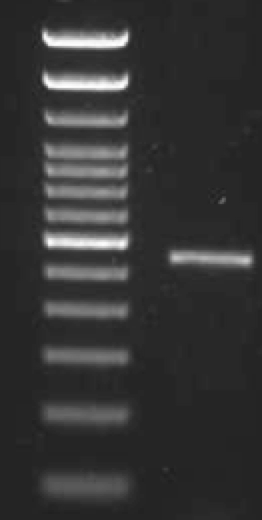


**A**


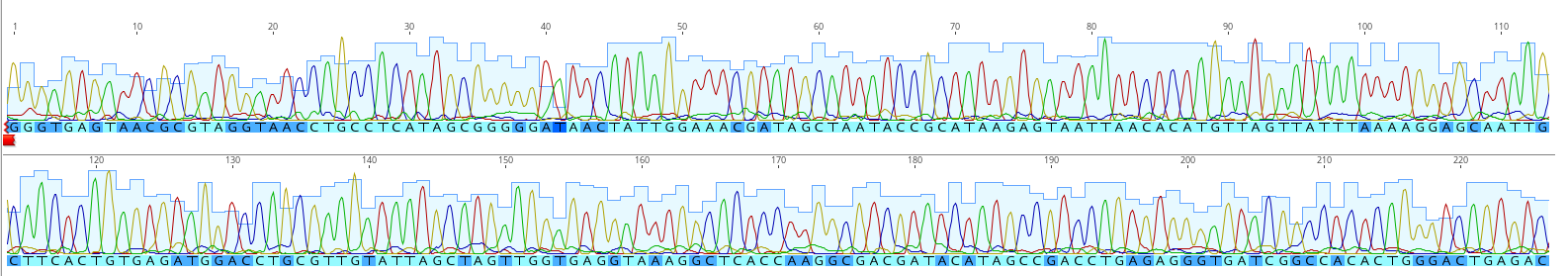


**B**

**C**


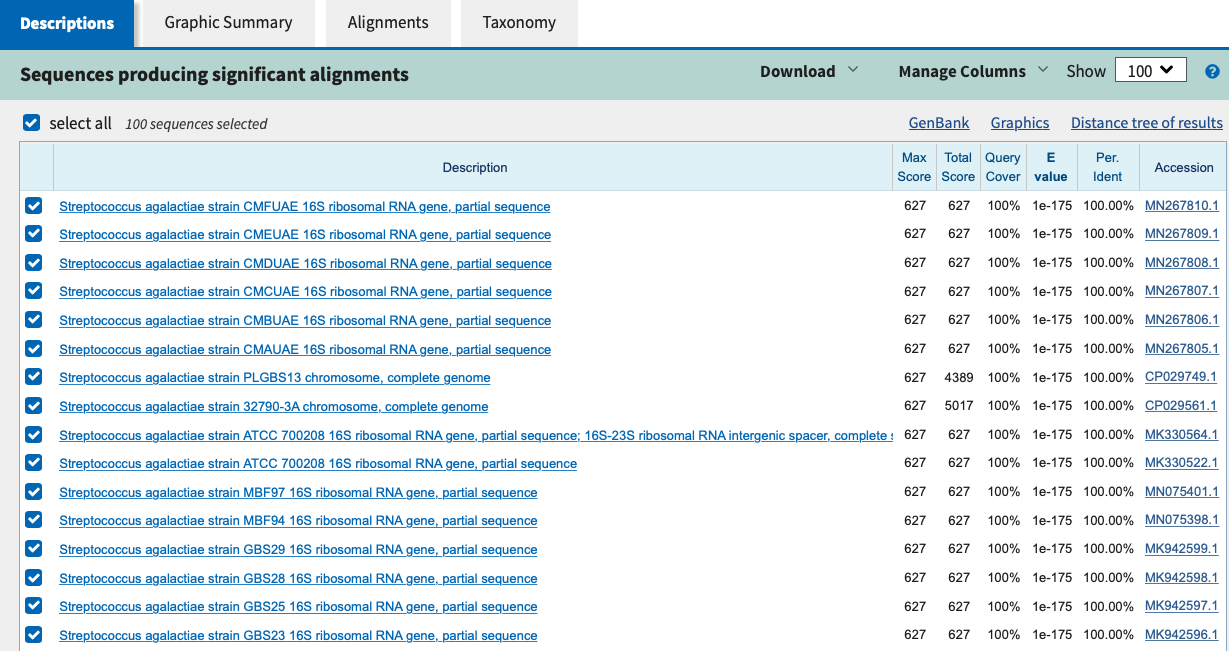


**Supplementary Figure 1:** Results of Sanger sequencing based analysis of 16S rRNA; A) Result of agarose gel analysis of the PCR product; B) A screen snapshot showing the result of Sanger sequencing; C) Top hits of the BLAST result of the obtained sequence

**Supplementary Figure 2**: Cumulative number of *S. agalacticiae* reads obtained after 25min, 50min, 75min and 100 min of the sequencing run.

**Supplementary Figure 3**: Flowchart showing an overview of the MinION sequencing of 16S rRNA gene

**DNA extraction from clinical sample**

**Library preparation**

16S Barcoding Kit (SQK-RAB204)

**MinION sequencing**

**and basecalling**

**Sequence analysis**

Barcode demultiplexing and

Pathogen identification (EPI2ME)

1 hour

3 hours

1-2 hours
